# Precision of MRI-based body composition measurements of postmenopausal women

**DOI:** 10.1371/journal.pone.0192495

**Published:** 2018-02-07

**Authors:** Janne West, Thobias Romu, Sofia Thorell, Hanna Lindblom, Emilia Berin, Anna-Clara Spetz Holm, Lotta Lindh Åstrand, Anette Karlsson, Magnus Borga, Mats Hammar, Olof Dahlqvist Leinhard

**Affiliations:** 1 Department of Medical and Health Sciences (IMH), Linköping University, Linköping, Sweden; 2 Center for Medical Image Science and Visualization (CMIV), Linköping University, Linköping, Sweden; 3 Advanced MR Analytics AB, Linköping, Sweden; 4 Department of Biomedical Engineering (IMT), Linköping University, Linköping, Sweden; 5 Department of Clinical and Experimental Medicine (IKE), Linköping University, Linköping, Sweden; Vanderbilt University, UNITED STATES

## Abstract

**Objectives:**

To determine precision of magnetic resonance imaging (MRI) based fat and muscle quantification in a group of postmenopausal women. Furthermore, to extend the method to individual muscles relevant to upper-body exercise.

**Materials and methods:**

This was a sub-study to a randomized control trial investigating effects of resistance training to decrease hot flushes in postmenopausal women. Thirty-six women were included, mean age 56 ± 6 years. Each subject was scanned twice with a 3.0T MR-scanner using a whole-body Dixon protocol. Water and fat images were calculated using a 6-peak lipid model including R2*-correction. Body composition analyses were performed to measure visceral and subcutaneous fat volumes, lean volumes and muscle fat infiltration (MFI) of the muscle groups’ thigh muscles, lower leg muscles, and abdominal muscles, as well as the three individual muscles pectoralis, latissimus, and rhomboideus. Analysis was performed using a multi-atlas, calibrated water-fat separated quantification method. Liver-fat was measured as average proton density fat-fraction (PDFF) of three regions-of-interest. Precision was determined with Bland-Altman analysis, repeatability, and coefficient of variation.

**Results:**

All of the 36 included women were successfully scanned and analysed. The coefficient of variation was 1.1% to 1.5% for abdominal fat compartments (visceral and subcutaneous), 0.8% to 1.9% for volumes of muscle groups (thigh, lower leg, and abdomen), and 2.3% to 7.0% for individual muscle volumes (pectoralis, latissimus, and rhomboideus). Limits of agreement for MFI was within ± 2.06% for muscle groups and within ± 5.13% for individual muscles. The limits of agreement for liver PDFF was within ± 1.9%.

**Conclusion:**

Whole-body Dixon MRI could characterize a range of different fat and muscle compartments with high precision, including individual muscles, in the study-group of postmenopausal women. The inclusion of individual muscles, calculated from the same scan, enables analysis for specific intervention programs and studies.

## Introduction

Body composition measurements are increasingly important for diagnosis and monitoring of metabolic diseases [1,2], muscle diseases [3] and metabolic components of other diseases [4–6]. Body composition refers to the compartmental distribution of fat and muscle within the body including visceral adipose tissue volume (VAT), abdominal subcutaneous adipose tissue volume (ASAT), liver-fat fraction, muscle group volumes such as thigh muscles, individual muscle volumes, and muscle fat infiltration (MFI). Several methods have been put forward to determine fat and muscle distribution in the body, such as dual-energy x-ray absorptiometry (DXA) [7] and bioimpedance (BIA) [8]. However, these methods do not allow direct quantification of absolute compartmental tissue volumes in a consistent and accurate manner. Novel magnetic resonance imaging (MRI) techniques, on the other hand, allow rapid body composition measurements with high precision and accuracy, which makes it possible to utilize the techniques in research and clinical applications.

Magnetic resonance imaging provides tomographic images with high soft tissue contrast, which enables quantification of fat and muscle compartmental volumes. Especially Dixon methods [9], that produce co-registered water and fat images, facilitate the separation of both adipose and lean tissue compartments. Scanning the whole body with sufficient spatial resolution is achievable in 6–8 minutes on modern scanner systems, using specialized body composition protocols available today [10, 11].

Recently, methods have been suggested that automatically or semi-automatically identify and quantify fat and muscle tissue volumes using MRI [12–16]. These methods measure body fat [12, 13, 17], or body muscle [14–16, 18, 19], but combined fat and muscle measurement methods have also been reported [10, 11]. Assessment of accuracy and precision of measurement of MFI using fat referenced Dixon imaging following manual segmentation has previously been validated by Peterson et al. [20]. Borga et al. [10] reported intra-observer coefficients of variation (CV) of 1.6% and 1.1% for quantification of VAT and abdominal subcutaneous adipose tissue (ASAT) respectively from abdominal MR images. The corresponding inter-observer CVs were 1.4% and 1.2% respectively. Test-retest reproducibility for whole body and compartmental muscle volumes were reported by Thomas et al. [16] with limits of agreement of -0.07–0.03 L and -0.08–0.04 L for right and left thigh muscles respectively. Newman et al. [21] similarly reported on test-retest precision for quantifying whole body and compartmental fat volumes with CVs of 1.80%, 2.98% and 0.79% and limits of agreement of -0.12–0.13 L, -0.55–0.64 L, and -0.67–0.60 L for VAT, ASAT, and total adipose tissue respectively. Middleton et al. [22] reported on accuracy and repeatability for the combined quantification of fat, muscle, and liver proton density fat-fraction (PDFF) with CVs of 1.5%– 3.6% for adipose tissue and thigh muscle volumes and intra- and inter-examination CVs of 6.5% and 7.3% respectively for liver PDFF.

While these studies show high reproducibility and accuracy for whole body measurements and groups of muscles, such as thigh muscles, it is challenging to combine this with smaller individual muscles based on the same MR-acquisition. To our knowledge, no study has previously reported on the reproducibility of individual muscle volumes combined with the more common whole body and compartmental measurements.

One subject group of particular interest is postmenopausal women, where fat-accumulation tend to shift toward increasing visceral adiposity. As a consequence of this, postmenopausal women display variability in phenotypes depending on their specific fat-accumulation pattern. Also, many women experience decreased quality of life *e*.*g*. due to hot flushes and sleep disturbances. A recent review concluded that increased weight in middle-aged women is mainly due to chronological aging while changed body composition and fat distribution after menopause are related to ovarian aging [23]. Increased body fat, especially central fat deposits, are related to both high cardiovascular risks and increased risk to develop cancer [24]. Increased MFI has been shown to correlate with functional outcomes in sarcopenia [25] and is commonly observed in investigations of type 2 diabetes [26]. Obese postmenopausal women have an increased breast cancer risk, probably mainly due to increased oestrogen production by adipose tissue from adrenal androgens, but also due to chronic adipose tissue inflammation [27]. Hot flushes and sweating affect about 75% of all women after menopause often causing sleep disturbances and decreased quality of life [28]. Exercise has been suggested as a treatment option to reduce hot flushes but studies have been inconclusive [29]. MRI-based body composition measurements are included in an on-going intervention study aiming to assess the effects of resistance training (RT) as a treatment alternative for hot flushes [30]. It will be important to assess the shift in fat compartments and the effect of the training on individual muscle volumes as a result of the intervention program. Determining baseline precision is important to assess dynamic changes during the longitudinal study. Measurement precision is essential in longitudinal intervention studies as the precision pose a lower limit on the detectable changes.

This was a sub-study of the larger randomized control trial (RCT) investigating effects of resistance training in postmenopausal women where the primary outcome is hot flush frequency. The aim of this study was **(1)** to determine precision for MRI-based fat and muscle measurements in the study-group at baseline, and **(2)** to extend the method to measure volume of specific individual muscles relevant to the main RCT training-program.

## Materials and methods

### Subjects

The main RCT (registered as ID: NCT01987778) is an open, parallel group, randomized controlled intervention study conducted in Linköping, Sweden. From the main RCT 36 postmenopausal women were enrolled and included in this precision sub-study. Subjects were enrolled on a voluntary basis, and all subjects that volunteered for MRI and had been included in the main RCT were included in this sub-study. Full details on inclusion/exclusion and the rationale for using resistance training as intervention have been reported in Berin et al. [30]; in summary sedentary women who were at least 45 years old and postmenopausal (*i*.*e*. more than 12 months since last menstrual bleeding) were invited to the study by means of advertisements in the local newspaper. Exclusion criteria were *e*.*g*. use of therapy that may influence hot flushes and physical activity more than 225 minutes per week of any intensity (including a maximum of 75 minutes per week of moderate to vigorous intensity). The study was performed according to the Declaration of Helsinki and Good Clinical Practice, and the study protocol was approved by The Regional Ethical Review Board in Linköping, Sweden (No: 2013/285-31). Written informed consent was obtained from all subjects prior to study entry.

### In-vivo imaging

In-vivo imaging was performed using a Philip Ingenia 3.0 T MR-scanner (Philips, Best, The Netherlands). Each subject was scanned twice on one occasion, where the subject was removed from the scanner room in between acquisitions. The protocol was a four-point 3D spoiled gradient multi-echo protocol with real and imaginary image reconstruction, acquired using a dStream WholeBody coil array. Total head-feet coverage was 1.76 m, divided over ten overlapping slabs of axial image with 25 mm overlap. Common parameters for all slabs were; flip-angle 10°, repetition time TR = 6.69 ms, echo times TE = 1.15/2.30/3.45/4.60 ms and voxel size 2.5×2.5×4 mm^3^. The first and last four slabs consisted of 66 slices; slabs two to six consisted of 39 slices accelerated using a SENSE factor of 1.6, acquired during 17-seconds expiration breath-holds. Additional abdominal slabs with a flip angle of 5°, but otherwise identical, were acquired over the liver for liver-fat quantification. Water and fat images were calculated from the multi echo images [31], using a 6-peak lipid model including R2*-correction [32]. Phase sensitive reconstruction was performed using an in-house MATLAB (The MatchWorks Inc. Natick, MA) implementation.

### Body composition analyses

Body composition analyses for abdominal fat and muscles were performed from the reconstructed water and fat images, using the commercially available service AMRA^®^ Profiler (Advanced MR Analytics AB, Linköping, Sweden). The methods used in AMRA^®^ Profiler have been thoroughly described in earlier publications [10, 11, 15, 33] but briefly the analysis consisted of the following steps: **(1)** image calibration to fat referenced images, **(2)** labels of fat and muscle compartments registered to the acquired volumes, **(3)** quality control of labels performed by trained analysis engineers at Advanced MR Analytics (Linköping, Sweden), and **(4)** quantification of fat and muscle volumes based on the calibrated images by integrating over the quality controlled labels. This process was described in detail in [11]. The included fat and muscle compartments were visceral adipose tissue (VAT), abdominal subcutaneous adipose tissue (ASAT), posterior thigh muscles, anterior thigh muscles, lower leg muscles, and abdominal muscles, detailed definitions of the anatomical regions used for compartmental fat and muscle segmentations and quality control are listed in Table 1. Finally, the individual muscles latissimus dorsi, pectoralis major, and rhomboideus were included.

**Table 1:** Anatomical definitions of fat and muscle compartments used for segmentations and quality control.

Anatomical Region	Definition
Visceral Adipose Tissue (VAT)	Adipose tissue within the abdominal cavity, excluding adipose tissue outside the abdominal skeletal muscles and adipose tissue and lipids within and posterior of the spine and posterior of the back muscles
Abdominal Subcutaneous Adipose Tissue (ASAT)	Subcutaneous adipose tissue in the abdomen from the top of the femoral head to the top of the thoracic vertebrae T9
Posterior Thigh Muscles	Gluteus muscles, iliacus, adductor muscles, and hamstring muscles
Anterior Thigh Muscles	Quadriceps femoris, sartorius, and tensor fascia latae
Lower Leg Muscles	All muscles between patella and talus
Abdominal Muscles	All muscles within the torso and neck, excluding muscles belonging to the arms

Muscle fat infiltration was measured for each muscle. The MFI measurements were defined as the average PDFF of the muscle tissue, *i*.*e*. muscle tissue with an adipose tissue concentration of less than 50%. As the calibrated fat images are T1-corrected [20], and represent the adipose tissue concentration of the tissue, the MFI was calculated by scaling the adipose tissue concentration with the PDFF of adipose tissue. In this study a constant PDFF of 93.7% was assumed for adipose tissue to convert adipose tissue concentration to PDFF.

Based on water-fat images acquired with a 5° flip angle, the liver-fat was measured as the average PDFF of three 22x22x28 mm^3^ regions of interest (ROI) manually placed in right liver lobe, avoiding major vessels and bile ducts. The liver test and re-test scans were pooled and analysed in randomized order.

### Statistical analysis

Descriptive statistics (mean ± SD) were calculated for all volumes. Precision was calculated as repeatability, defined in [34], using Bland-Altman analysis (mean of difference, and limits of agreement). The repeatability conditions were the same measurement procedure, same operators, same MR-scanner, same location, and replicate measurements over a short period of time. The within-subject standard deviation, *sw*, was estimated as the square root of the mean within-subject variance. The repeatability was calculated as 2.77 * *sw*, as suggested by Bland and Altman [35]. This definition of ‘repeatability’ was based on the assumption that the difference between any two measurements of the same subject is expected to be less than this for 95% of pairs of observations. Systematic effects were investigated with paired t-tests between the first and second scan. Finally, the within-subject CV was calculated. Statistical analysis was performed in R version 3.4.0 (2017-04-21, The R Foundation).

## Results

**Fig 1:**
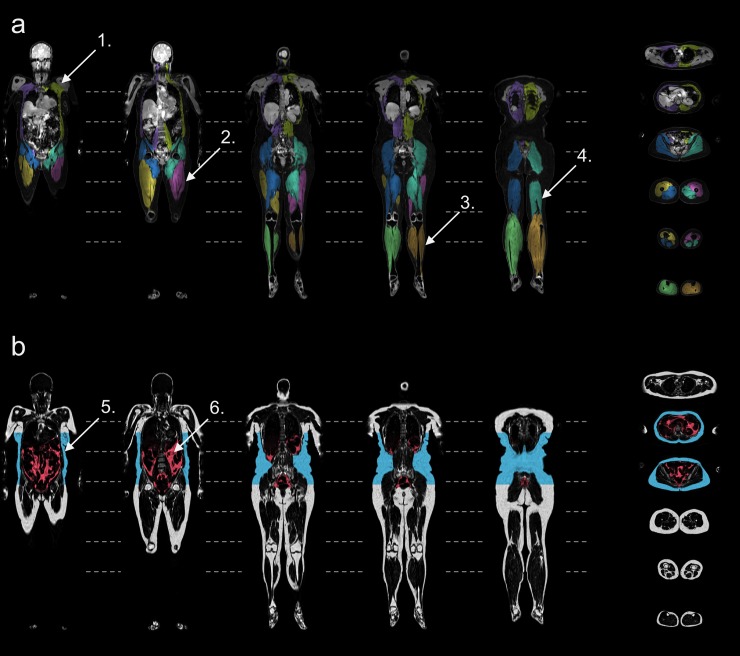
Segmentation of fat and muscle compartments. Sample compartmental segmentation result, coronal and transverse sections of a 61-years-old woman. **(a)** Intensity-corrected water images with muscle segmentations using overlay colours, 1. abdominal muscles, 2. anterior thigh, 3. lower leg muscles, 4. posterior thigh, **(b)** intensity-corrected fat images with fat segmentations using overlay colours, 5. abdominal subcutaneous adipose tissue, 6. visceral adipose tissue.

**Fig 2:**
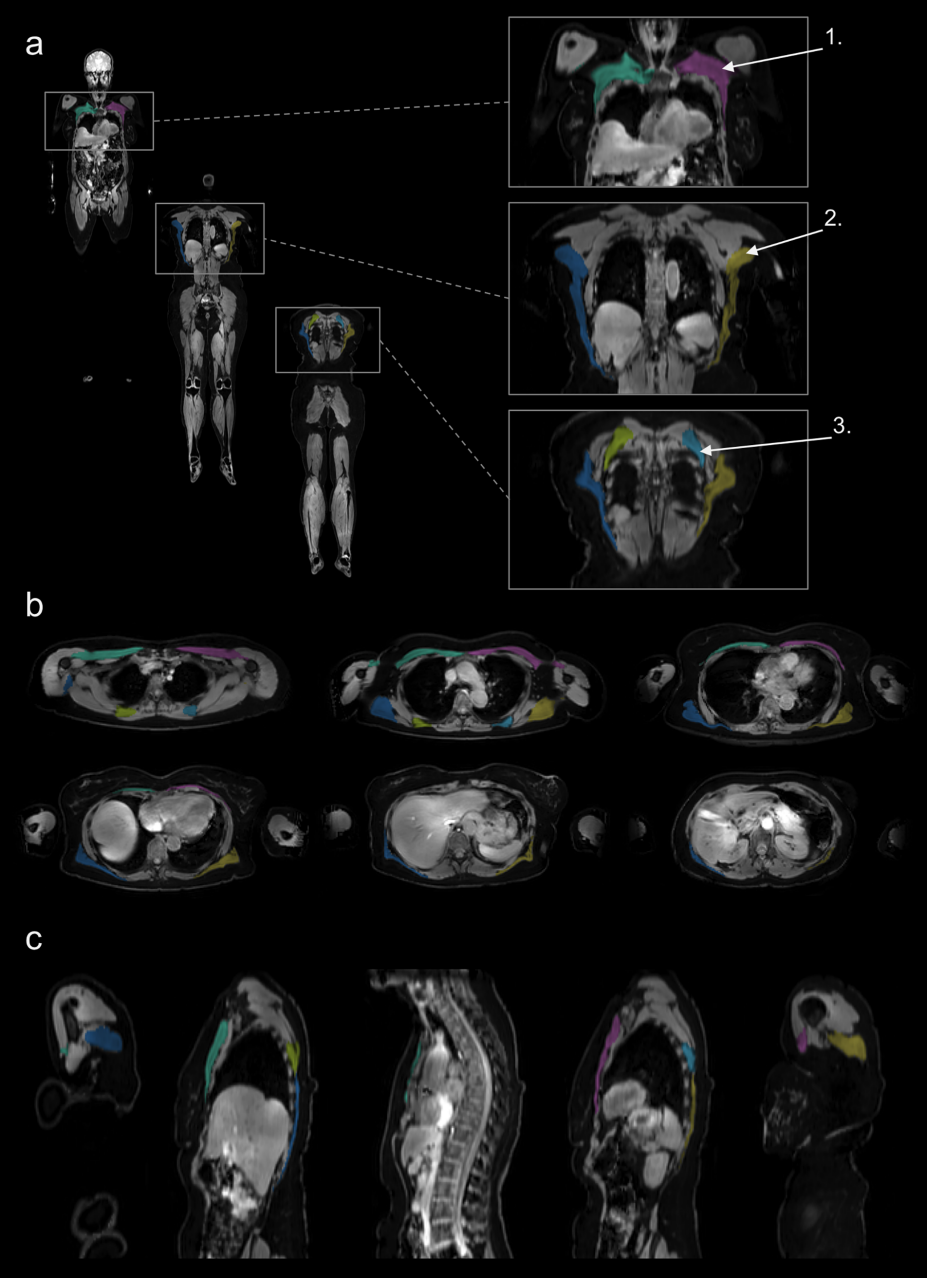
Segmentation of individual muscles. Sample individual muscle segmentation results of a 61-years-old woman. **(a)** Intensity-corrected water images with muscle segmentations using overlay colours, enlarged cutouts of central abdominal region 1. pectoralis major, 2. latissimus dorsi, 3. rhomboideus, **(b)** transverse sections covering the central abdominal region with muscle segmentations using overlay colours, and **(c)** sagittal cutouts of the central abdominal region with muscle segmentations using overlay colours.

**Fig 3:**
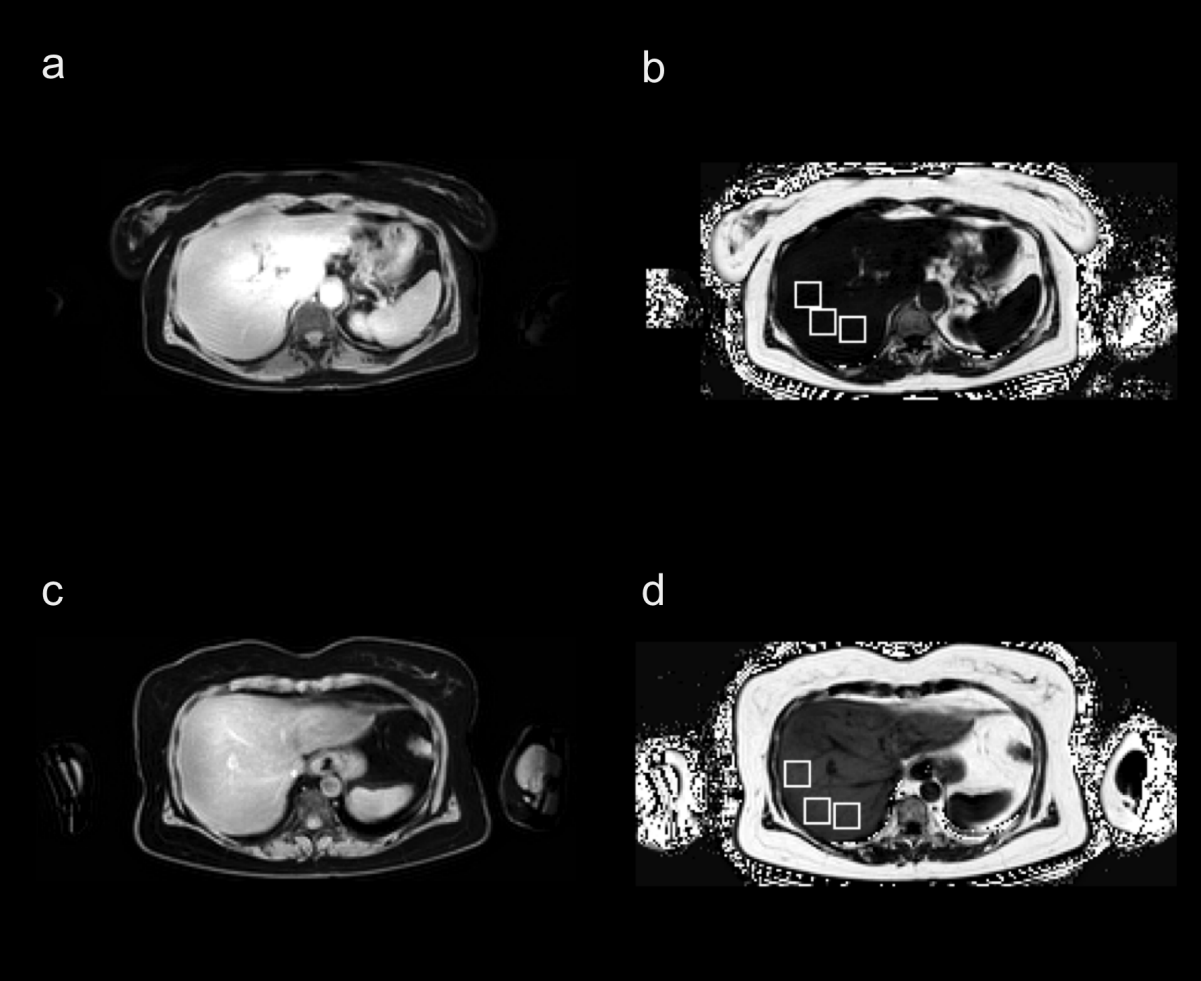
Region of interest placements in the liver. Water and proton density fat-fraction (PDFF) liver images with region of interest (ROI) placements to assess liver-fat, in **(a-b)** a subject with low liver-fat content, and **(c-d)** a subject with higher liver-fat content. **(a, c)** Water images and **(b, d)** PDFF images.

All of the 36 included women were scanned and analysed with approved quality control, as defined in [11], including *e*.*g*. that no slabs were missing and no severe swaps were present. The mean age was 56 ± 6 years (range 45 to 70 years), the mean BMI was 26.5 ± 3.6 kg/m^2^ (range 18.9 to 33.5 kg/m^2^). Acquisition time for each scan was 8:00 minutes. Representative results for the compartmental fat and muscle segmentations are shown in Fig 1, and representative results for the individual muscle segmentations are shown in Fig 2 with enlarged regions covering the abdominal area. Sample ROI placement used to assess liver-fat is shown in Fig 3.

Within the group, the mean VAT was 2.50 ± 1.30 L (range 0.73 L to 5.93 L), mean ASAT was 8.30 ± 2.74 L (range 2.61 L to 12.84 L), mean total thigh volume was 8.92 ± 1.18 L (range 6.80 L to 10.94 L), and mean total thigh MFI was 9.02 ± 1.98% (range 6.02 to 13.00%). Complete volumes and liver fat details are reported in Table 2, and MFI are reported in Table 3.

**Table 2:** Descriptive statistics and ranges for volumes and liver fat.

Measurement	Mean	SD	Min	Max
Liver fat (%)	3.92	5.51	-1.85	26.23
VAT (L)	2.50	1.30	0.73	5.93
ASAT (L)	8.30	2.74	2.60	12.84
Left Posterior Thigh (L)	2.91	0.37	2.24	3.59
Right Posterior Thigh (L)	2.90	0.38	2.19	3.59
Left Anterior Thigh (L)	1.52	0.24	0.99	2.05
Right Anterior Thigh (L)	1.60	0.25	1.13	2.17
Total Thigh (L)	8.92	1.18	6.80	10.94
Left Lower Leg (L)	1.43	0.22	0.98	1.84
Right Lower Leg (L)	1.44	0.24	1.02	1.86
Left Abdomen (L)	2.67	0.30	2.13	3.47
Right Abdomen (L)	2.69	0.28	2.16	3.41
Left Latissimus (L)	0.231	0.035	0.158	0.291
Right Latissimus (L)	0.254	0.039	0.172	0.330
Left Pectoralis (L)	0.197	0.029	0.116	0.242
Right Pectoralis (L)	0.212	0.029	0.134	0.256
Left Rhomboideus (L)	0.0266	0.0062	0.0084	0.0403
Right Rhomboideus (L)	0.0291	0.0070	0.0092	0.0429

Abbreviations; visceral adipose tissue (VAT), abdominal subcutaneous adipose tissue (ASAT), standard deviation (SD).

**Table 3:** Descriptive statistics and ranges for muscle fat infiltration.

Measurement	Mean	SD	Min	Max
Left Posterior Thigh MFI (%)	11.66	2.32	7.94	15.63
Right Posterior Thigh MFI (%)	10.10	2.29	6.14	14.26
Left Anterior Thigh MFI (%)	7.83	1.88	4.92	12.29
Right Anterior Thigh MFI (%)	6.51	1.68	3.63	10.45
Total Thigh MFI (%)	9.03	1.98	6.02	13.00
Left Lower Leg MFI (%)	8.19	1.88	4.85	12.20
Right Lower Leg MFI (%)	6.36	1.87	3.89	11.05
Left Abdomen MFI (%)	14.57	2.57	9.95	20.17
Right Abdomen MFI (%)	13.78	2.65	9.25	19.69
Left Latissimus MFI (%)	13.73	2.64	9.33	20.58
Right Latissimus MFI (%)	12.32	2.63	7.66	19.27
Left Pectoralis MFI (%)	12.46	3.24	7.84	20.79
Right Pectoralis MFI (%)	11.18	2.99	4.98	19.07
Left Rhomboideus MFI (%)	12.27	2.23	8.79	16.49
Right Rhomboideus MFI (%)	9.89	2.74	5.40	14.63

Abbreviations; muscle fat infiltration (MFI), abdominal subcutaneous adipose tissue (ASAT), standard deviation (SD).

The repeatability was 0.116 L for VAT, 0.346 L for ASAT, and 0.246 L for total thigh volume, and 1.32% for total thigh MFI. Furthermore, the repeatability was 1.69% for liver fat, 0.0477 L to 0.186 L for the larger muscle regions, and 0.0054 L to 0.0220 L for the individual muscles. Complete volumes and liver fat precision statistics are reported in Table 4, and MFI precision statistics are reported in Table 5.

**Table 4:** Precision statistics for muscle and fat volumes as well as liver fat.

Measurement	sw	Repeatability	CV (%)	Limits of agreement lower bound	Limits of agreement upper bound
Liver fat (%)	0.61	1.69	18.68%	-1.91	1.32
VAT (L)	0.0417	0.116	1.54%	-0.127	0.103
ASAT (L)	0.125	0.346	1.06%	-0.385	0.291
Left Posterior Thigh (L)	0.0415	0.115	1.01%	-0.127	0.100
Right Posterior Thigh (L)	0.0367	0.102	0.87%	-0.113	0.085
Left Anterior Thigh (L)	0.0172	0.0477	0.90%	-0.0542	0.0322
Right Anterior Thigh (L)	0.0170	0.0472	0.87%	-0.0533	0.0364
Total Thigh (L)	0.0889	0.246	0.75%	-0.279	0.184
Left Lower Leg (L)	0.0273	0.0757	1.43%	-0.0860	0.0494
Right Lower Leg (L)	0.0211	0.0585	1.09%	-0.0664	0.0385
Left Abdomen (L)	0.0671	0.186	1.88%	-0.183	0.194
Right Abdomen (L)	0.0607	0.168	1.79%	-0.176	0.164
Left Latissimus (L)	0.0072	0.0200	2.25%	-0.0179	0.0219
Right Latissimus (L)	0.0080	0.0220	2.39%	-0.0201	0.0239
Left Pectoralis (L)	0.0063	0.0173	2.57%	-0.0173	0.0178
Right Pectoralis (L)	0.0069	0.0191	2.68%	-0.0188	0.0199
Left Rhomboideus (L)	0.0019	0.0054	6.03%	-0.0056	0.0053
Right Rhomboideus (L)	0.0025	0.0069	6.98%	-0.0069	0.0071

Within-subject standard deviation (sw), repeatability, coefficient of variation (CV), and Bland-Altman limits of agreement for all measurements. Abbreviations; visceral adipose tissue (VAT), abdominal subcutaneous adipose tissue (ASAT).

**Table 5:** Precision statistics for muscle fat infiltration.

Measurement	sw	Repeatability	CV (%)	Limits of agreement lower bound	Limits of agreement upper bound
Left Posterior Thigh MFI (%)	0.45	1.25	2.82%	-1.27	1.26
Right Posterior Thigh MFI (%)	0.53	1.46	3.99%	-1.38	1.56
Left Anterior Thigh MFI (%)	0.59	1.64	5.79%	-1.37	1.83
Right Anterior Thigh MFI (%)	0.65	1.81	7.49%	-1.29	2.06
Total Thigh MFI (%)	0.48	1.32	3.88%	-1.12	1.46
Left Lower Leg MFI (%)	0.45	1.26	4.59%	-1.30	1.26
Right Lower Leg MFI (%)	0.53	1.47	6.37%	-1.47	1.52
Left Abdomen MFI (%)	0.44	1.21	2.46%	-1.35	1.05
Right Abdomen MFI (%)	0.42	1.17	2.31%	-1.33	0.84
Left Latissimus MFI (%)	0.68	1.90	3.88%	-2.07	1.71
Right Latissimus MFI (%)	0.62	1.73	4.15%	-1.81	1.69
Left Pectoralis MFI (%)	1.33	3.68	8.30%	-4.10	3.10
Right Pectoralis MFI (%)	1.73	4.80	12.49%	-5.13	4.55
Left Rhomboideus MFI (%)	0.76	2.10	4.94%	-2.30	1.89
Right Rhomboideus MFI (%)	0.83	2.31	7.04%	-2.30	2.39

Within-subject standard deviation (sw), repeatability, coefficient of variation (CV), and Bland-Altman limits of agreement for all measurements. Abbreviations; visceral adipose tissue (VAT), abdominal subcutaneous adipose tissue (ASAT), muscle fat infiltration (MFI).

**Fig 4:**
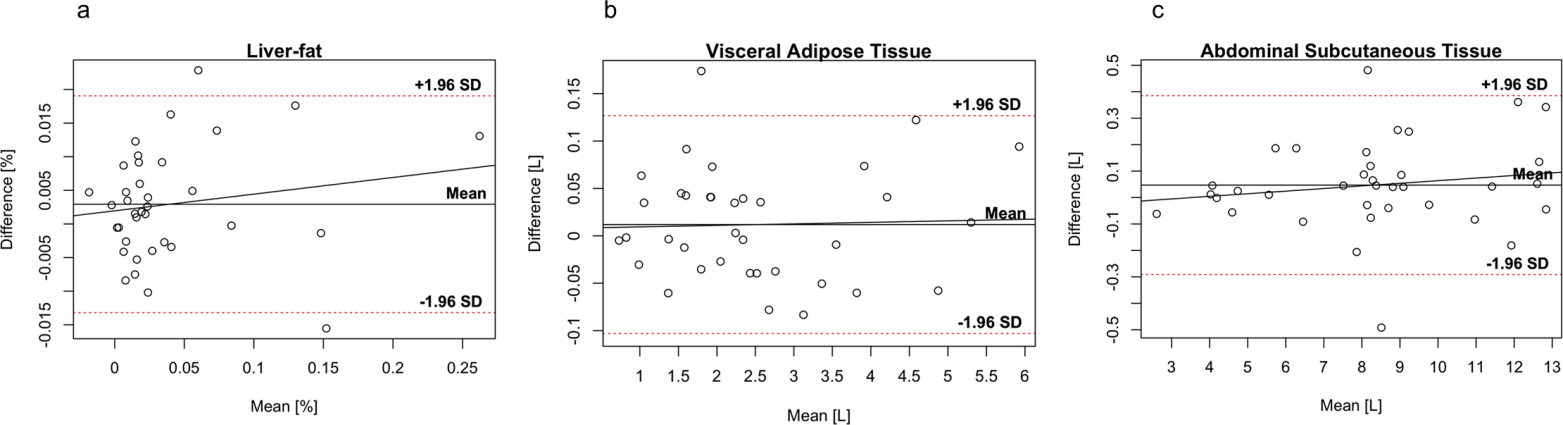
Bland-Altman plots of fat compartments. Bland-Altman plots of **(a)** liver-fat, **(b)** visceral adipose tissue, and **(c)** abdominal subcutaneous adipose tissue. Note: Different ranges on y-axes.

**Fig 5:**
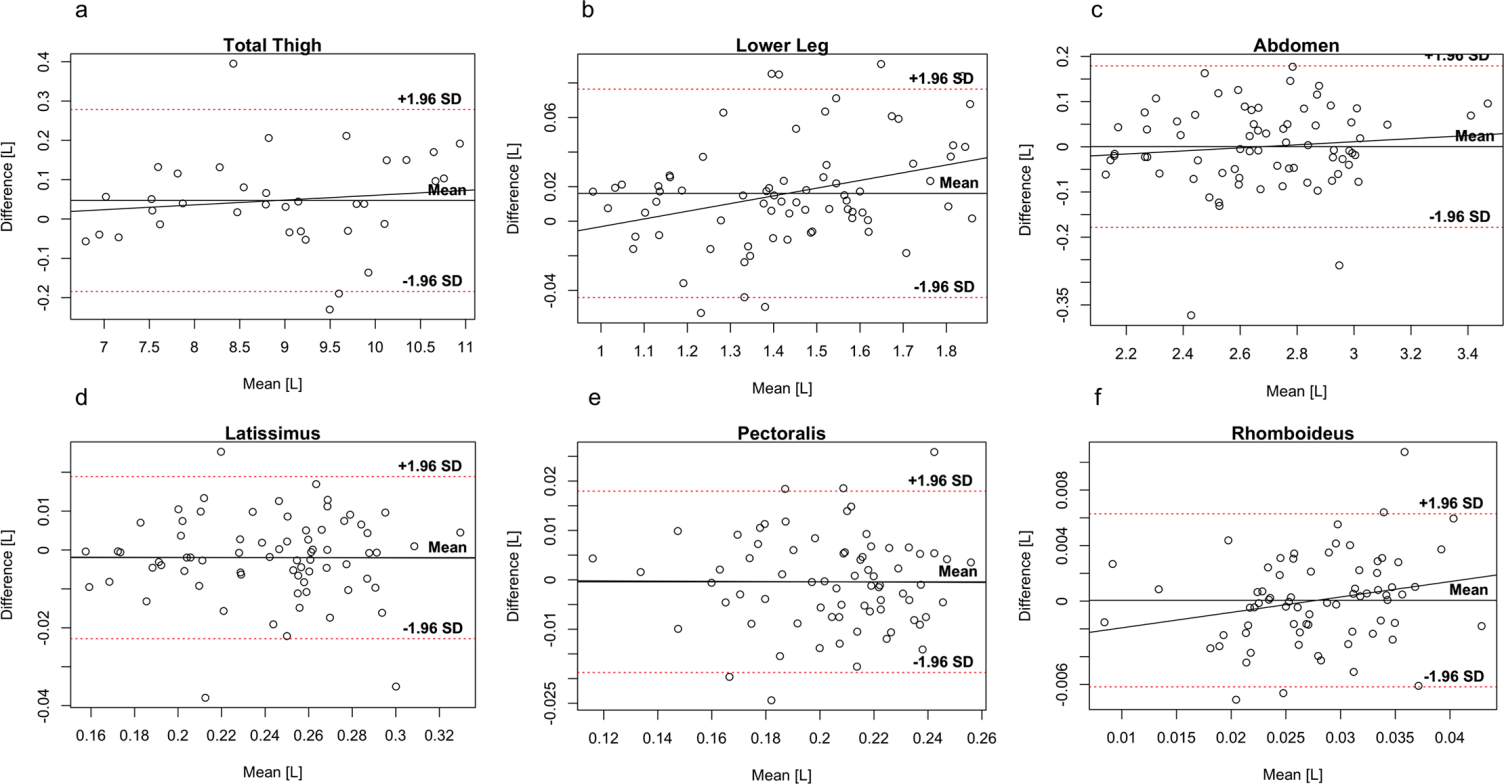
Bland-Altman plots of muscle compartments and individual muscles. Bland-Altman plots of **(a)** total thigh, **(b)** lower leg, **(c)** abdomen, **(d)** latissimus, **(e)** pectoralis, and **(f)** rhomboideus. For bilateral muscles **(b-f)** left and right sides are pooled in the plots. Note: Different ranges on y-axes.

**Fig 6:**
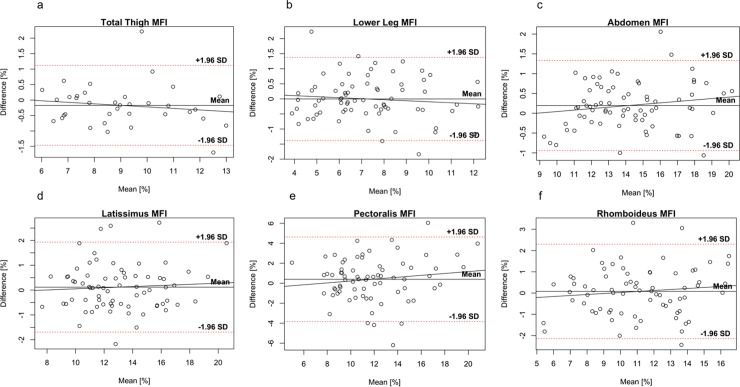
Bland-Altman plots of muscle fat infiltration (MFI). Bland-Altman plots of **(a)** total thigh, **(b)** lower leg, **(c)** abdomen, **(d)** latissimus, **(e)** pectoralis, and **(f)** rhomboideus. For bilateral muscles **(b-f)** left and right sides are pooled in the plots. Note: Different ranges on y-axes.

Bland-Altman plots for fat compartments are shown in Fig 4, and for the muscle groups and individual muscles in Fig 5, and for MFI in Fig 6. Paired t-tests revealed no systematic differences between the first and second scan, p-value range 0.675 to 0.995.

The complete body composition measurements results as provided by AMRA^®^ Profiler are available in the supporting information file S1 Table.

## Discussion

This study was a baseline sub-study to an on-going RCT, with the purpose of determining precision for MRI-based compartmental fat and muscle measurements, and to extend the measurements to specific individual muscles that are relevant to the main RCT training-program. Body composition measurements were calculated from a single rapid Dixon-based MR acquisition including the volume of VAT, ASAT, and PDFF in the liver, as well as the fat-free muscle volume and MFI in the posterior thigh, anterior thigh, lower leg, and muscles in the abdomen. Furthermore the individual muscles latissimus dorsi, pectoralis major, and rhomboideus were included.

In this study, whole-body coverage was achieved using an MR protocol with ten slabs and a voxel size of 2.5×2.5×4 mm^3^. The protocol was similar to previously published results from the vast population study in UK Biobank [11]. In that study, the success rate for body composition analyses was very high (99.83% for body fat and 92.50% for body fat and all included muscle compartments), and for the present study all women could be fully analysed. The women were scanned with a four-point Dixon protocol that had to be reconstructed to fat- and water-separated images using an in-house MATLAB implementation. The reason for this was that the analysis software only supported natively reconstructed images and not four-point Dixon images. In this study, we found a CV of 1.54% for VAT and 1.06% for ASAT, which was similar to previously published results by Borga et al. [10] (VAT CV 1.6% and ASAT CV 1.1%) and results reported by Newman et al. [21] (ASAT CV 2.98%), but lower than results reported by Middleton et al. [22] who reported a VAT CV of 3.3% and ASAT CV of 2.2%. The limits of agreement for total thigh muscle volume of -0.28–0.18 L was somewhat higher than previously reported limits of agreement by Thomas et al. [16] (total thigh limits of agreement -0.07–0.03 L (right leg) and -0.08–0.04 L (left leg)). One reason for this could be differences in the MRI protocols, where Thomas et al. used a higher resolution over the thighs. Individual muscle measurements with this method have not been reported previously. In general, smaller muscles and liver fat measurements suffer from higher CV, as can be expected due to the lower volumes. When the mean value is close to zero, as for liver fat in this cohort, the CV will approach infinity and will become sensitive to small changes in the mean. We found CV in the range of 2.25% - 2.68% for the largest muscles up to 6.03% - 6.98% for the smallest muscle. The range of limits of agreement for the precision of measured MFI was lower than ±2% for all larger muscles in the study. Only the MFI of the smaller muscles rhomboideus and pectoralis was higher which is not unexpected given the limited image resolution. It should be noted that a mean systematic right/left difference of 1.5% (range 0.8% to 2.4%) was observed in the average MFI. This right/left asymmetry is probably associated to scan related artefacts such as gradient system performance or reconstruction filters but seems to be stable in between examinations as the limits of agreement of the precision was lower than ± 1.5% for several of the larger muscles.

The measurements in this sub-study were made at baseline of the main RCT, *i*.*e*. these women were not allowed physical activity more than 225 minutes per week of any intensity, whereof maximum 75 minutes per week moderate or vigorous intensity. The main RCT is studying the effect of resistance training as a treatment alternative for hot flushes, where the training program includes upper-body exercise. It is important to assess the changes in fat compartments, the overall effect on muscles, and the change in specific muscles following the intervention program, compared to a control group. There are two reasons that the specific muscles latissimus, pectoralis, and rhomboides were included. First, these muscles are relevant in upper-body exercise. Second, the three muscles are generally in different volume regimes, where latissimus is a large muscle (in this group 0.23 ± 0.04 L for left and 0.25 ± 0.04 for right), pectorialis is normally smaller (0.20 ± 0.03 left, 0.21 ± 0.03 right), and rhomboideus is the smallest muscle (0.03 ± 0.01 left, 0.03 ± 0.01 right). This encompasses a representative sample of muscle sizes and this may be extrapolated to measurements of other muscles in different intervention programs, and studies. Furthermore, the women presented a diversity of phenotypes, especially concerning the visceral adipose tissue (range 0.73–5.97 L) and abdominal subcutaneous adipose tissue (range 2.58–13.00 L), as can be expected due to the shift in fat-accumulation pattern during menopause.

### Limitations

In this MR-study we used a standardized MR-protocol for whole-body coverage and body composition analysis. The protocol was optimized to balance scan-time and acquired volume in terms of coverage, voxel size and signal-to-noise-ratio (SNR). In this study, the acquisition resolution and SNR was too low to achieve high precision for the smallest muscle, rhomboideus. As a consequence of this the results showed higher CV for this muscle. One method to increase the resolution and SNR could be to add additional breath-hold slabs, but this would also increase the scan-time. Furthermore, all individual muscles that were measured in this study were in the abdominal region that is affected by breathing artefacts, with a 17-second breath-hold time it is possible that the reported precision was somewhat affected by this. While sufficient for all subjects in this particular study, the maximum height for head-feet coverage was 1.76 m. In a different study population adding an additional slab, at the expense of scan-time, could increase this.

A further limitation of this study was that all data was collected at baseline. Although this gives a comprehensive view of the baseline precision, in terms of repeatability, it is not possible to assess the effects of daily variations, that are likely to affect the precision and required power in the main RCT. Also, it was not possible to assess reproducibility where the experimental conditions change, such as a different MR-scanner or different operators.

## Conclusions

In conclusion, this study verifies that whole-body Dixon MRI can characterize a range of different fat and muscle compartments with high precision in the study-group of postmenopausal women. Furthermore, the method was successfully extended to allow precise measurements of individual muscles. The results support the use of combined fat and muscle measurements on a compartmental level and for individual muscles, based on a single rapid MR acquisition. The addition of individual muscle measurements, calculated from the same scan, opens the possibility to tailor the analysis for specific intervention programs and studies.

## Supporting information

S1 Table Complete body composition measurements results. Columns in the file are, for the first scan: liver PDFF (liver_fat_t), visceral adipose tissue (vat_t), abdominal subcutaneous adipose tissue (asat_t), left posterior thigh muscle volume (lp_thigh_t), right posterior thigh muscle volume (rp_thigh_t), left anterior thigh muscle volume (la_thigh_t), right anterior thigh muscle volume (ra_thigh_t), total thigh muscle volume (total_thigh_t), left lower leg muscle volume (l_lower_leg_t), right lower leg muscle volume (r_lower_leg_t), left abdominal muscle volume (l_abd_t), right abdominal muscle volume (r_abd_t), left latissimus muscle volume (l_lat_t), right latissimus muscle volume (r_lat_t), left pectoralis muscle volume (l_pec_t), right pectoralis muscle volume (r_pec_t), left rhomboideus muscle volume (l_rho_t), right rhomboideus muscle volume (r_rho_t), and for the second scan: liver PDFF (liver_fat_r), visceral adipose tissue (vat_r), abdominal subcutaneous adipose tissue (asat_r), left posterior thigh muscle volume (lp_rhigh_r), right posterior thigh muscle volume (rp_rhigh_r), left anterior thigh muscle volume (la_rhigh_r), right anterior thigh muscle volume (ra_rhigh_r), total thigh muscle volume (total_rhigh_r), left lower leg muscle volume (l_lower_leg_r), right lower leg muscle volume (r_lower_leg_r), left abdominal muscle volume (l_abd_r), right abdominal muscle volume (r_abd_r), left latissimus muscle volume (l_lat_r), right latissimus muscle volume (r_lat_r), left pectoralis muscle volume (l_pec_r), right pectoralis muscle volume (r_pec_r), left rhomboideus muscle volume (l_rho_r), right rhomboideus muscle volume (r_rho_r). MFI measurements are prefixed by “mfi_” followed by the muscle or muscle group and test/retest as defined above.
(XLSX)
